# Natural variation of physiological traits, molecular markers, and chlorophyll catabolic genes associated with heat tolerance in perennial ryegrass accessions

**DOI:** 10.1186/s12870-020-02695-8

**Published:** 2020-11-16

**Authors:** Jing Zhang, Hui Li, Yiwei Jiang, Huibin Li, Zhipeng Zhang, Zhipeng Xu, Bin Xu, Bingru Huang

**Affiliations:** 1 College of Agro-grassland Science, Nanjing Agricultural University, Nanjing, 210095 P.R. China; 2 Department of Agronomy, Purdue University, West Lafayette, IN 47907 USA; 3 College of Agronomy, Hebei Agricultural University/State Key Laboratory of North China Crop Improvement and Regulation/Key Laboratory of Crop Growth Regulation of Hebei Province, Baoding, 071001 Hebei China; 4 Shanghai Biotechnology Corporation, Zhangjiang Hi-tech Park, Shanghai, 201203 P.R. China; 5 Department of Plant Biology and Pathology, Rutgers, the State University of New Jersey, New Brunswick, NJ 08901 USA

**Keywords:** Perennial ryegrass, Leaf senescence, Heat tolerance, SSR, Genetic diversity

## Abstract

**Background:**

Identification of genetic diversity in heat tolerance and associated traits is of great importance for improving heat tolerance in cool-season grass species. The objectives of this study were to determine genetic variations in heat tolerance associated with phenotypic and physiological traits and to identify molecular markers associated with heat tolerance in a diverse collection of perennial ryegrass (*Lolium perenne* L.).

**Results:**

Plants of 98 accessions were subjected to heat stress (35/30 °C, day/night) or optimal growth temperature (25/20 °C) for 24 d in growth chambers. Overall heat tolerance of those accessions was ranked by principal component analysis (PCA) based on eight phenotypic and physiological traits. Among these traits, electrolyte leakage (EL), chlorophyll content (Chl), relative water content (RWC) had high correlation coefficients (− 0.858, 0.769, and 0.764, respectively) with the PCA ranking of heat tolerance. We also found expression levels of four Chl catabolic genes (CCGs), including *LpNYC1*, *LpNOL*, *LpSGR*, and *LpPPH*, were significant higher in heat sensitive ryegrass accessions then heat tolerant ones under heat stress. Furthermore, 66 pairs of simple sequence repeat (SSR) markers were used to perform association analysis based on the PCA result. The population structure of ryegrass can be grouped into three clusters, and accessions in cluster C were relatively more heat tolerant than those in cluster A and B. SSR markers significantly associated with above-mentioned traits were identified (*R*^*2*^ > 0.05, *p* < 0.01)., including two pairs of markers located on chromosome 4 in association with Chl content and another four pairs of markers in association with EL.

**Conclusion:**

The result not only identified useful physiological parameters, including EL, Chl content, and RWC, and their associated SSR markers for heat-tolerance breeding of perennial ryegrass, but also highlighted the involvement of Chl catabolism in ryegrass heat tolerance. Such knowledge is of significance for heat-tolerance breeding and heat tolerance mechanisms in perennial ryegrass as well as in other cool-season grass species.

## Background

Improving heat tolerance is among major efforts of breeding improvement in cool-season grass species [1]. The available germplasm collections with large genetic variability and a wide range of heat-tolerance levels are important breeding materials for grass breeding program [2–4]. To make good use of the germplasm pool, it is essential to understand their genetic diversity as well as physiological and molecular factors underlying heat stress tolerance for the breeding of heat-tolerant cultivars.

Understanding the genetic structure and phenotypic diversity using phenotypic traits and molecular markers is the basis of parental selection for trait improvement in breeding, such as for heat tolerance [5–7]. Phenotypic analysis focuses on growth, morphological, and physiological parameters among germplasm and provides information regarding genetic diversity, homogeneity, and stability [7, 8]. Since abiotic stress adversely affect a multitude of morphological and physiological processes, a number of distinct morphological and physiological characteristics have been used as marker traits to evaluate genetic variations in plant stress tolerance, such as root activity (RA), photochemical efficiency (Fv/Fm), photosynthetic rate (Pn), water use efficiency (WUE), chlorophyll (Chl) content, and leaf relative water content (RWC) [7, 9–11]. The selection of germplasm based on physiological traits is an efficient approach in breeding for improved stress tolerance in various crop species [7, 12–14]. In the case of cool-season grass breeding for heat tolerance, such information of closely associated physiological trait(s) to heat tolerance is still largely unclear.

Molecular markers linked to phenotypic and physiological traits have been developed to understand the genetic diversity and to predict desirable traits of a given germplasm or breeding materials [7]. To date, molecular makers associated with several important agronomic traits of perennial ryegrass have been developed, including crown rust resistance [15], drought tolerance [16], winter survival and spring re-growth [17], submergence [18], and salinity tolerance (Tang et al., 2013). For examples, Yu et al. (2011) evaluated the submergence tolerance of 99 diverse perennial ryegrass accessions using 109 simple sequence repeat (SSR) markers, and identified 15 pairs of SSR markers associated with alterations of several morphological and physiological traits (e.g. leaf color, Fv/Fm, maximum plant height, and relative growth rate) [18]. Tang et al. (2013) analyzed the genetic diversity of 56 perennial ryegrass accessions of different origins using 66 SSR makers, and found that population structure influenced phenotypic traits, and allelic variation in *LpNHX1* might explain the variation of salinity tolerance of perennial ryegrass [19]. Yet, molecular markers associated with heat-tolerant are to be developed in perennial cool-season grass species.

Perennial ryegrass (*Lolium perenne* L.), native to Europe, Asia, and Northern Africa, is the most widely cultivated perennial cool-season grass in the temperate regions worldwide for its turf and forage purposes [20, 21]. Perennial ryegrass is considered as a heat-sensitive grass species, and the established ryegrass sward is often challenged by heat stress. Despite of the diversity of perennial ryegrass to various other stresses and diseases, as discussed above, neither the diversity of heat tolerance among perennial ryegrass germplasm collections was quantitatively measured, nor the molecular markers associated with heat tolerance been developed for this species. The objectives of this study were to understand physiological traits and to identify molecular markers associated with heat tolerance in a diverse collection of perennial ryegrass. Such knowledge will be valuable for heat-tolerant breeding in perennial ryegrass as well as other cool-season grass species.

## Results

### Evaluation of heat tolerance in 98 accessions of perennial ryegrass

A total of seven physiological traits (WUE, Pn, RA, Chl content, RWC, EL, and Fv/Fm) and three phenotypic traits (TQ, LW, and PH) were used to evaluate heat tolerance of 98 perennial ryegrass accessions (Supplementary materials 1). Effects of heat stress treatment, genotype, and interaction of these two factors were all significant (*p* ≤ 0.05) for WUE, Pn, RA, Chl, RWC, Fv/Fm, EL, and TQ. Only genotypic variations were significant for LW and PH (Table 1).

**Table 1:** Summary of variance for the effects of treatments, genotypes, and the interaction between temperature and genotype on photochemical efficiency (Fv/Fm), chlorophyll content (Chl), photosynthesis rate (Pn), water use efficiency (WUE), electrolyte leakage (EL), leaf relative water content (RWC), root activity (RA), turf quality (TQ), leaf width (LW), and plant height (PH) across 98 perennial ryegrass accessions with the data of 24 days treatment

	Physiological traits							Morphological traits		
	Fv/Fm	Chl	Pn	WUE	EL	RWC	RA	TQ	LW	PH
Treatment	***	***	***	***	***	***	***	***	NS	NS
Genotype	*	**	**	**	*	**	**	**	*	**
Treatment ╳ Genotype	**	***	***	***	**	***	**	***	NS	NS

**Fig. 1:**
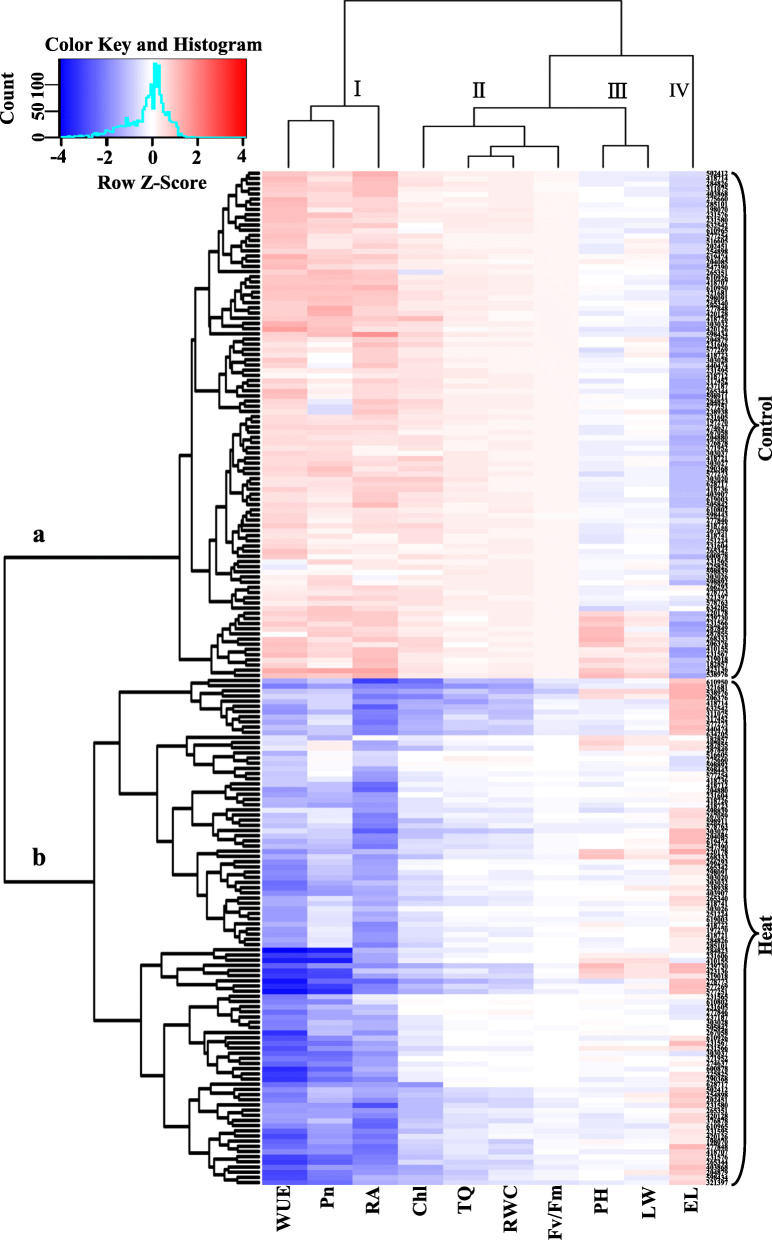
Heatmap and hierarchical clustering for physiological and morphological parameters under control and heat stress conditions in 98 ryegrass accessions after 24 d of treatment. Abbreviations: WUE, water use efficiency; Pn, photosynthesis rate; RA, root activity; Chl, chlorophyll content; RWC, leaf relative water content; Fv/Fm, photochemical efficiency; PH, maximum plant height; LW, leaf width

The data for physiological and phenotypic traits of 98 accessions exposed to heat stress or optimum temperature (control) conditions were used to plot a heatmap (Fig. 1), in which the hierarchical cluster was clearly grouped into two distinct sub-clusters: accessions under the control condition (sub-cluster ‘a’) and those under the heat stress (sub-cluster ‘b’ as shown in Fig. 1). Accordingly, ten traits used to evaluate ryegrass heat tolerance were grouped into four sub-clusters: sub-cluster I included WUE, Pn, and RA, where their values were lower under heat stress than those under control condition; sub-cluster II included Chl content, TQ, RWC, and Fv/Fm, which values were also lower under heat stress; sub-cluster III, including the two morphological traits (PH and LW), did not show consistent alteration under the two growth conditions; and sub-cluster IV, only consisted of one trait (EL), had higher values under heat stress (Fig. 1). Therefore, eight traits, excluding LW and PH, were used to evaluate ryegrass heat tolerance in the following analyses.

### Ranking of overall heat tolerance for 98 accessions of perennial ryegrass

The PCA analysis of variations in heat tolerance of 98 accessions based on HSI identified a total of eight principal components (PC 1–8). The sum of the first PCs (PC1 to PC4) explained 88.45% of the total variance, among which the 1st and 2nd PCs were the major ones explaining 52.67 and 20.37% of the variance among 98 ryegrass accessions, respectively (Supplementary Table 1). Based on the PCA result, the following formulas were developed (details of the formula were shown in Supplementary Table 2): PC1 value = 0.897 × TQ + 0.766 × Fv/Fm + 0.858 × Chl content + 0.373 × Pn + 0.244 × WUE + (− 0.691) × EL + 0.891 × RWC + 0.785 × RA; and PC2 value = (− 0.199) × TQ + (− 0.311) × Fv/Fm + (− 0.055) × Chl content + 0.809 × Pn + 0.896 × WUE + (− 0.043) × EL + (− 0.180) × RWC + 0.040 × RA; (3) PC3 value = 0.196 × TQ + 0.413 × Fv/Fm + (− 0.119) × Chl content + 0.233 × Pn + 0.033 × WUE + 0.566 × EL + 0.095 × RWC + (− 0.227) × RA; and PC4 value = 0.031 × TQ + (− 0.125) × Fv/Fm + 0.219 × Chl content + (− 0.220) × Pn + 0.130 × WUE + 0.431 × EL + (− 0.166) × RWC + 0.480 × RA, and PCA rank value = (52.67% × PC1) + (20.37% × PC2) + (8.25% × PC3) + (7.17% × PC4).

**Fig. 2:**
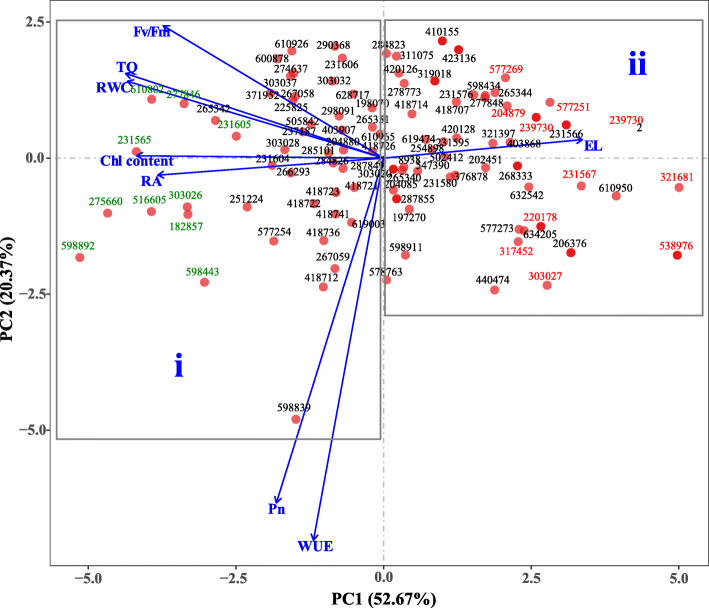
Principal component analysis biplot of the heat stress index (HSI) of 98 ryegrass accessions. *Arrows* represent physiological and morphological traits with various lengths based on the impact of each trait on the separation of accessions. Accessions marked with green color in group i and red color in group ii are the ten most and least heat tolerant genotypes, respectively. Abbreviations: WUE, water use efficiency; Pn, photosynthesis rate; RA, root activity; Chl, chlorophyll content; RWC, leaf relative water content; Fv/Fm, photochemical efficiency; PH, maximum plant height; LW, leaf width

**Fig. 3:**
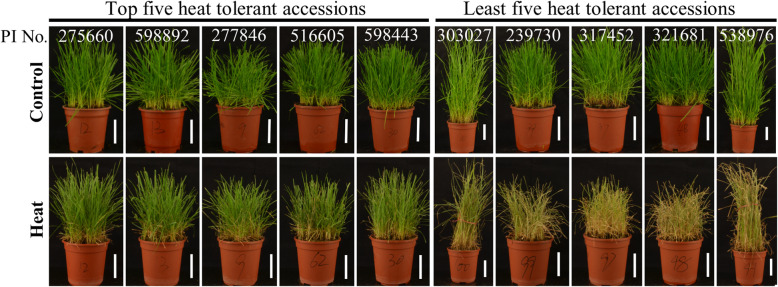
Phenotype of the five most and least heat tolerant ryegrass accessions. Pictures were taken after 24 d of treatment. Bar in each photo represents 6.5 cm in length

According to PCA results, we clustered 98 ryegrass accessions into two groups:Group-i consisted of 49 accessions with PCA rank value at the top half of all accessions, while the rest 49 accessions clustered to group-ii (Fig. 2). Heat tolerance of 98 accessions were then ranked according to their PCA ranking values based on the HIS of eight parameters (WUE, Pn, RA, Chl content, RWC, and Fv/Fm, EL, and TQ), with accessions 275,660, 598,892, 277,846, 516,605, and 598,443 ranked as the top five accessions for heat tolerance and 538,976, 321,681, 317,452, 239,730, and 303,027 ranked as the most heat-sensitive accessions (Table 2). Phenotypes of the top and least five heat tolerant ryegrass accessions were shown in Fig. 3, that plants of the top-rated accessions had more green leaves or greener leaves while the most heat-sensitive accessions had more yellow or less green leaves.

**Table 2:** The four major components (PC1, PC2, PC3, and PC4) and PCA ranking of each ryegrass genotypes based on turf quality (TQ), photochemical efficiency (Fv/Fm), chlorophyll content (Chl), photosynthesis rate (Pn), water use efficiency (WUE), electrolyte leakage (EL), leaf relative water content (RWC), and root activity (RA)

Accession No.	PC1	PC2	PC3	PC4	PCA value	Rank
275,660	299.1	10.4	128.3	50.7	173.95	1
598,892	282.5	16.2	142.3	84.7	169.99	2
277,846	268.9	−28.5	109.0	46.4	148.22	3
516,605	242.4	9.7	156.2	75.7	148.06	4
598,443	239.6	31.2	140.9	49.6	147.79	5
610,802	261.1	−33.9	118.7	75.9	145.94	6
231,565	245.7	−19.2	136.5	95.8	143.69	7
303,026	194.6	0.9	169.4	101.1	123.97	8
231,605	207.8	−20.7	140.7	71.7	122.03	9
182,857	171.5	−3.3	188.3	116.8	113.60	10
265,342	184.8	−26.5	177.2	96.3	113.53	11
251,224	170.8	5.3	180.9	80.8	111.81	12
577,254	160.9	16.0	182.5	80.1	108.85	13
231,604	167.9	−13.3	163.7	90.9	105.78	14
266,293	167.9	−11.2	160.2	77.9	104.98	15
598,839	129.8	60.5	187.4	109.5	104.04	16
303,037	182.0	−43.0	135.9	70.6	103.42	17
418,722	157.7	2.0	162.7	67.4	101.77	18
303,028	149.0	−14.4	180.1	83.8	96.43	19
237,187	146.3	−23.3	167.8	82.4	92.11	20
418,712	117.3	20.0	193.7	100.2	89.07	21
371,952	140.9	−41.5	183.4	109.4	88.79	22
267,058	137.9	−34.3	184.0	96.4	87.80	23
284,826	132.4	−11.1	172.7	73.1	87.02	24
505,842	135.1	−27.4	178.8	88.2	86.68	25
600,878	140.6	−49.0	176.7	103.7	86.10	26
418,736	114.5	10.1	197.9	91.7	85.29	27
274,637	137.6	−45.1	179.2	95.3	84.92	28
285,101	124.5	−13.6	185.9	81.5	84.03	29
578,763	109.4	19.2	170.4	81.5	81.49	30
225,825	111.7	−37.7	202.4	115.7	76.18	31
619,003	98.0	4.3	199.2	99.5	76.07	32
298,091	113.7	−30.4	181.0	93.0	75.31	33
418,741	97.6	−0.5	192.6	108.4	74.99	34
418,726	114.5	−22.4	163.6	79.6	74.99	35
418,723	98.6	−9.3	200.4	108.4	74.38	36
610,965	115.6	−26.8	156.0	81.2	74.17	37
267,059	80.9	19.7	225.7	112.7	73.38	38
403,907	101.8	−27.3	189.6	101.0	70.95	39
204,880	90.8	−21.1	207.6	105.2	68.21	40
610,926	99.2	−53.4	212.1	122.8	67.69	41
418,721	82.3	−6.4	209.3	104.9	66.85	42
265,351	88.4	−31.8	187.3	109.6	63.40	43
231,606	92.2	−50.6	197.8	101.9	61.90	44
303,020	75.8	−15.7	187.1	94.7	58.97	45
198,070	83.0	−25.4	168.9	88.7	58.85	46
628,717	76.7	−39.8	207.7	102.3	56.77	47
306,292	73.4	−43.5	215.0	125.5	56.55	48
290,368	75.4	−55.9	214.5	121.1	54.73	49
265,340	56.2	−8.7	207.8	114.2	53.17	50
287,855	42.8	−4.7	242.3	132.5	51.09	51
197,270	25.1	−1.8	262.8	143.8	44.85	52
502,412	44.7	−17.8	194.8	102.1	43.29	53
284,823	50.2	−54.1	213.9	125.3	42.07	54
634,205	39.2	1.7	158.3	87.2	40.30	55
238,938	13.5	−16.6	274.8	153.7	37.42	56
321,397	42.2	−29.2	167.4	101.4	37.39	57
231,576	43.0	−42.0	178.8	100.6	36.06	58
231,595	24.1	−27.5	215.9	119.6	33.46	59
319,018	32.6	−45.5	203.5	116.1	32.99	60
420,126	23.3	−50.2	238.8	140.2	31.79	61
254,898	13.3	−22.7	223.5	133.6	30.39	62
202,451	11.9	−16.4	205.8	113.2	27.99	63
268,333	4.0	−14.2	235.3	127.4	27.76	64
598,434	24.7	−41.1	189.3	102.4	27.61	65
287,849	−12.4	−19.8	270.1	157.8	23.04	66
278,773	17.2	−29.3	158.4	84.2	22.20	67
547,390	−13.2	−19.6	262.5	159.2	22.11	68
231,580	1.3	−23.9	203.9	118.4	21.13	69
440,474	−33.7	21.5	253.8	141.0	17.69	70
204,085	−26.5	−20.6	284.0	165.4	17.13	71
403,868	−11.3	−28.7	216.3	126.5	15.09	72
598,911	−56.4	11.4	321.2	179.9	11.99	73
376,878	−41.5	−18.3	272.5	159.7	8.33	74
420,128	−33.3	−34.1	259.2	155.7	8.07	75
418,714	−49.3	2.5	234.9	133.0	3.47	76
418,707	−46.7	− 41.8	271.7	163.4	0.96	77
231,566	−53.4	−49.2	304.0	189.2	0.45	78
410,155	−38.5	−62.2	267.8	153.9	0.17	79
619,474	−61.4	−31.3	304.9	184.6	−0.36	80
577,273	−85.5	−3.0	284.4	170.6	−9.95	81
632,542	−81.0	−13.6	272.1	163.7	−11.27	82
265,344	−82.3	−47.0	283.7	182.6	−16.47	83
311,075	−93.3	−17.7	257.2	160.0	−20.10	84
206,376	− 116.3	1.2	284.7	174.5	−25.06	85
277,848	−109.2	−48.9	316.5	203.2	−26.84	86
423,136	− 108.7	−63.1	323.2	211.5	−28.32	87
610,950	−113.6	−13.9	260.8	153.3	−30.17	88
220,178	− 137.9	−45.4	377.7	250.5	−32.80	89
204,879	−123.2	−45.0	317.4	199.0	−33.66	90
577,251	−121.2	−45.1	294.4	184.2	−35.60	91
577,269	− 129.7	−55.1	324.2	209.2	−37.87	92
231,567	− 148.4	−65.1	398.0	261.8	−39.87	93
303,027	− 174.1	5.5	350.1	226.2	−45.53	94
239,730	− 151.2	− 42.2	330.8	214.0	−45.67	95
317,452	−187.5	−1.8	385.2	239.0	−50.24	96
321,681	−151.0	−20.3	258.6	168.4	−50.31	97
538,976	−290.2	−10.9	399.8	278.6	− 102.20	98

To understand contributions of different traits to heat tolerance in perennial ryegrass, the relationships between PCA ranking based on HSI for overall heat tolerance and each physiological/phenotypic trait was determined using Pearson correlation analysis. As shown in Table 3, the PCA ranking was significantly correlated with all eight traits used in the evaluation (*p* ≤ 0.05), among which ranking values of PCA and EL had the largest correlation coefficient (*r* = − 0.858). And ranking values of Chl content, RWC, and TQ also showed high correlation coefficients with those of PCA (*r* = 0.769, 0.764, and 0.744, respectively). Values of Pn and WUE had low correlation coefficients with those of PCA (*r* = 0.306 and − 0.216, respectively), although their correlations were statistically significant as well (*p* ≤ 0.05) (Table 3).

**Table 3:** Pearson correlation coefficients analysis among the value of turf quality (TQ), photochemical efficiency (Fv/Fm), chlorophyll content (Chl), photosynthesis rate (Pn), water use efficiency (WUE), electrolyte leakage (EL), leaf relative water content (RWC), root activity (RA) under heat stress conditions across 98 perennial ryegrass accessions

	PCA rank value	TQ	Fv/Fm	Chl	Pn	WUE	EL	RWC	RA
PCA rank values	1								
TQ	0.744^***^	1							
Fv/Fm	0.578^***^	0.750^***^	1						
Chl	0.769^***^	0.814^***^	0.671^***^	1					
Pn	0.304^**^	0.132	0.093	0.237^*^	1				
WUE	−0.216^*^	−0.097	0.109	0.264^**^	0.259^**^	1			
EL	−0.858^***^	−0.757^***^	−0.611^***^	−0.700^***^	−0.163	0.207^*^	1		
RWC	0.764^***^	0.865^***^	0.729^***^	0.793^***^	0.099	−0.143	−0.807^***^	1	
RA	0.502^***^	0.563^***^	0.459^***^	0.565^***^	0.073	−0.124	−0.411^***^	0.517^***^	1

^*^, ^**^ and ^***^ indicate significance at *P* < 0.05, *P* < 0.01 and *P* < 0.001, respectively

### Transcript levels of chlorophyll-catabolic genes correlated to heat tolerance in perennial ryegrass

**Fig. 4:**
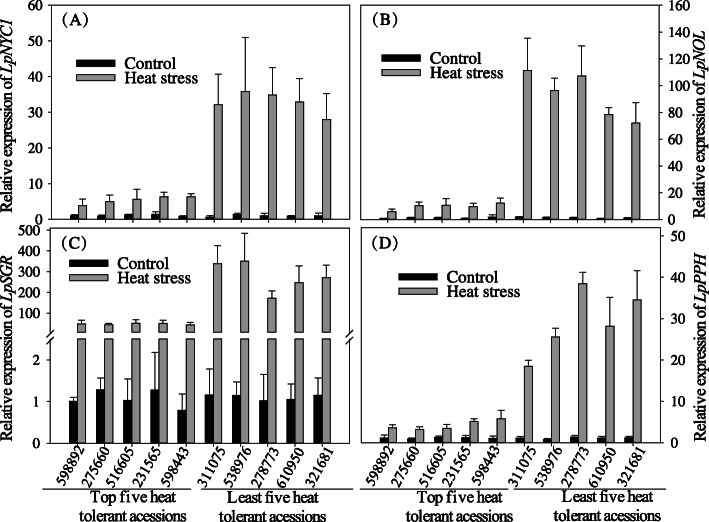
Relative expression levels of four CCGs of the five most and least heat-tolerant ryegrass accessions. Relative expression levels of *LpNYC1* (**a**), *LpNOL* (**b**), *LpSGR* (**c**), and *LpPPH* (D). Represented data were means and standard error (*n* = 4)

Results of Pearson correlation analysis indicated that heat tolerance PCA ranking values and Chl content had the larger correlation coefficient (*r* = 0.769, Table 3), indicating leaf senescence characterized by Chl loss was mostly associated with overall heat tolerance in perennial ryegrass. To confirm the contribution of Chl catabolism to heat-tolerant accessions of perennial ryegrass, we further analyzed whether there was a correlation between transcription of four Chl catabolic genes (*CCG*s, including *LpNYC1*, *LpNOL*, *LpSGR*, and *LpPPH*) and the PCA ranking values of heat tolerance of 98 ryegrass accessions. As shown in Fig. 4, relative expression levels of *CCG*s were significantly higher in heat-sensitive accessions than those in heat-tolerant accessions.

### Classification of 98 accessions of perennial ryegrass based on SSR markers

**Fig. 5:**
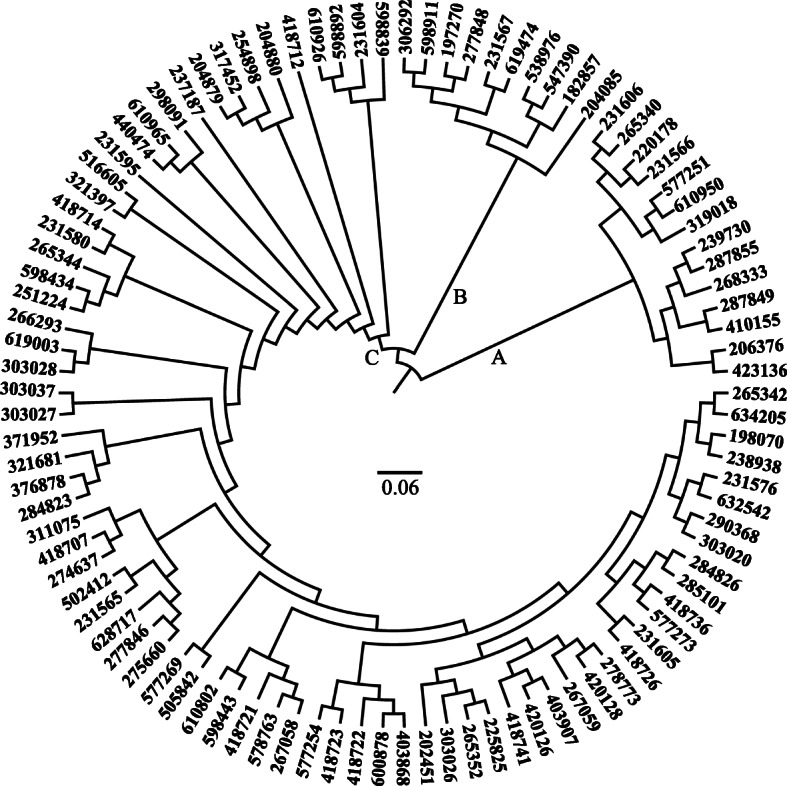
Neighbour-joining tree of 98 perennial ryegrass accessions based on SSR markers

Genotypic diversity within the selected ryegrass accessions was estimated using 66 pairs of SSR molecular markers. The SSR analysis yielded 864 polymorphic bands in total, with an average of 13 and a range of 3 to 26 bands *per* pair of primers (Supplementary material 2). The resultant polymorphism information content (PIC) values varied from 0.16 to 0.93, with an average of 0.70; while the gene diversity index (Di) values ranged 0.16 to 0.94, with an average of 0.72 (Supplementary materials 2), confirming that the selected accessions represented a diverse genetic pool of perennial ryegrass germplasm. An N-J dendrogram was constructed based on the SSR results, clustering the 98 ryegrass accessions into three groups: Cluster A, B, and C consisting of 14, 10, and 74 ryegrass accessions, respectively (Fig. 5).

Values of PCA ranking of heat tolerance and physiological traits were averaged across ryegrass accessions in each phylogenetic cluster (Table 4), and the results showed that averaged PCA ranking values of accessions in cluster C (61.67) were significantly higher than those in clusters A and B (2.65 and 7.89, respectively), suggesting that accessions in clusters A and B were less heat tolerant than those in cluster C. Similar difference was also observed for Chl content, WUE, EL, and RA for genotype ranking of heat tolerance in each phylogenetic cluster (Table 4).

**Table 4:** The average value of turf quality (TQ), photochemical efficiency (Fv/Fm), chlorophyll content (Chl, mg/g DW), photosynthesis rate (Pn, μmol CO_2_/m^2^/s), water use efficiency (WUE, μmol CO_2_/mmol H_2_O), electrolyte leakage (EL, %), leaf relative water content (RWC, %), root activity (RA, mg/g·h), and PCA ranking value of perennial ryegrass accessions in three phylogenetic clusters under heat stress condition

cluster	TQ	Fv/Fm	Chl	Pn	WUE	EL	RWC	RA	PCA
A	4.41	0.65	6.11b	3.76	0.67b	62.21a	57.91b	219.50b	2.65b
B	4.97	0.64	7.23ab	3.83	0.65b	65.17a	60.440ab	223.91b	7.89b
C	5.05	0.65	7.72a	3.77	0.77a	49.60b	63.44a	234.71a	61.67a

### SSR markers associated with physiological traits in heat tolerance

**Fig. 6:**
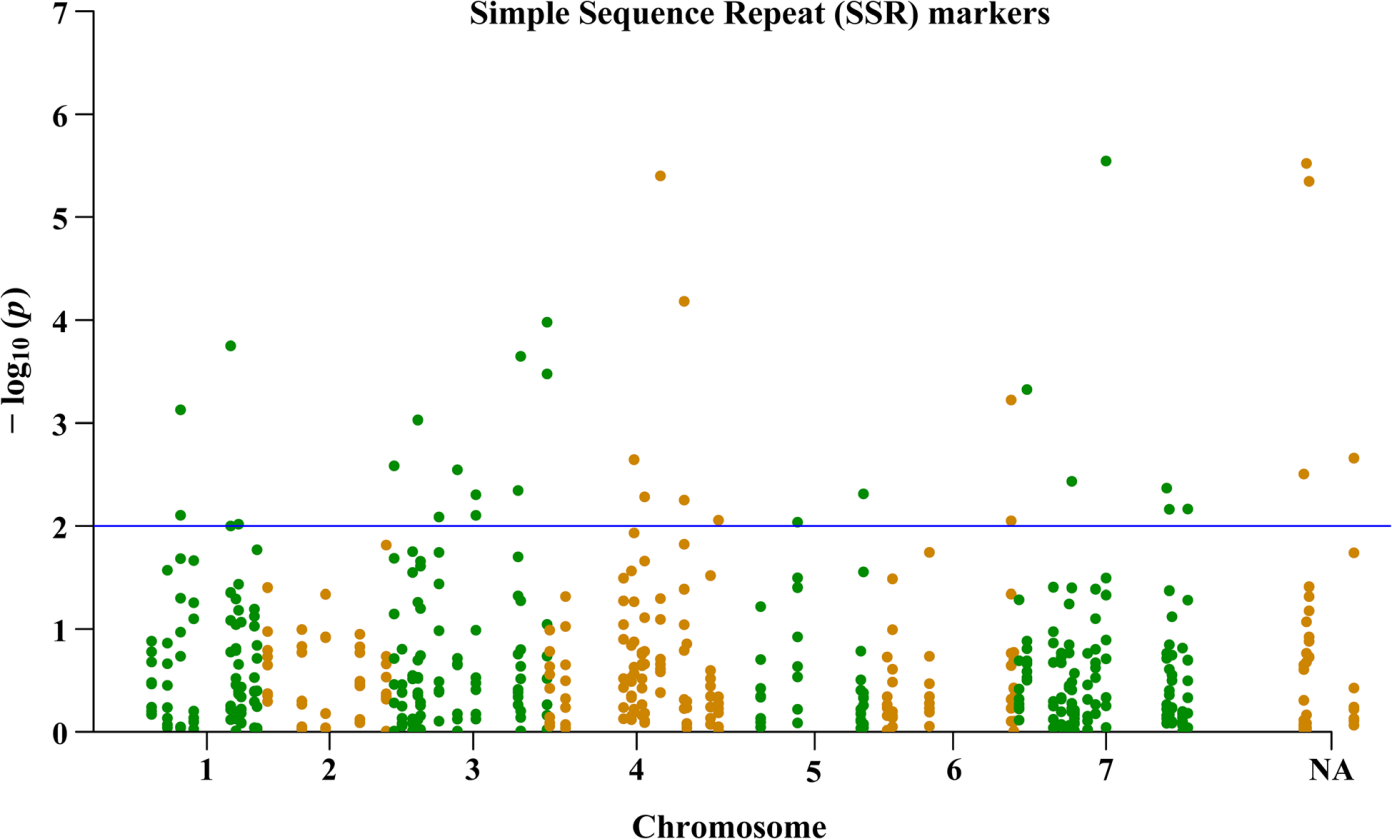
Manhattan plots of the general linear model (GLM) for association analysis between SSR markers and each physiological trait. The –log_10_(*P*-values) from each SRR markers are plotted against eight heat tolerance-related traits, including TQ, Fv/Fm, Chl content, Pn, WUE, RWC, RA, and EL. NA, not known

The associations between the 66 SSR markers and the seven physiological traits were further analyzed using a general linear model (GLM) in TASSEL. As shown in Fig. 6 and Table 5, a total of 34 associations were identified between the SSR markers and the relative values of Chl content, Fv/Fm, Pn, WUE, EL, and RA at *R*^*2*^ > 0.05 (*p* < 0.01). We found that two markers M144 and rv0941, located on chromosome 4, were associated with Chl content. The markers Lp165, rv0941, DLF008, B3C10, B3B8, and B5E1, located on chromosomes 3, 4, 5, and 7, were associated with Fv/Fm. The marker rv0985–1, located on chromosome 6, was associated with Pn. Thirteen markers, including PRG, PR10, M4213, 25ca1, LPSSRH01A07, rv0985–1, rv0005, rv1133, LPSSRH02C11, rv0663, B3B7, LpHCA16B2, and PR37, located on chromosomes 1, 3, 4, 6, and 7, were associated with WUE. The markers M844, LPSSRH01A07, B3B7, B1A10, LpSSR100, LP194, rv0757, and LPSSRH02C11, located on chromosomes 1, 3, and 5, were associated with RA. The markers LM15, LPSSRH01H06, rye012, and LpHCA17C6 were associated with EL. No association was identified between SSR markers and TQ or RWC (Fig. 6).

**Table 5:** Association of SSR markers with the heat stress index of Fv/Fm, Chl content, Pn, WUE, RA, and EL

Trait	Locus	Chromosome No.	Position (cM)	***P*** value	***R***^***2***^
**Fv/Fm**	LP165	7	66	2.85E-06	0.67
**Fv/Fm**	rv0941	4	20.3	6.57E-05	0.47
**Fv/Fm**	DLF008	7	77	4.74E-04	0.49
**Fv/Fm**	B3C10	7	80	3.68E-03	0.29
**Fv/Fm**	B3B8	3	70	8.18E-03	0.48
**Fv/Fm**	B5E1	5	NA	9.20E-03	0.19
**Chl**	M144	4	56	5.21E-03	0.52
**Chl**	rv0941	4	20.3	5.60E-03	0.37
**Pn**	rv0985–1	6	51.7	8.92E-03	0.64
**WUE**	PRG	4	119	3.98E-06	0.53
**WUE**	PR10	NA	NA	4.50E-06	0.47
**WUE**	M4213	1	46	1.78E-04	0.44
**WUE**	25ca1	3	91.1	2.25E-04	0.56
**WUE**	LPSSRH01A07	3	NA	3.34E-04	0.33
**WUE**	rv0985–1	6	51.7	5.99E-04	0.70
**WUE**	rv0005	7	0	4.28E-03	0.75
**WUE**	rv1133	3	27.8	4.52E-03	0.17
**WUE**	LPSSRH02C11	3	NA	4.97E-03	0.37
**WUE**	rv0663	7	6.7	6.88E-03	0.31
**WUE**	B3B7	1	49	7.87E-03	0.29
**WUE**	LpHCA16B2	4	0	8.79E-03	0.48
**WUE**	PR37	1	52	9.62E-03	0.48
**RA**	M844	NA	NA	3.01E-06	0.60
**RA**	LPSSRH01A07	3	NA	1.05E-04	0.35
**RA**	B3B7	1	49	7.47E-04	0.35
**RA**	B1A10	3	74	9.35E-04	0.63
**RA**	LpSSR100	3	62	2.60E-03	0.21
**RA**	LP194	NA	NA	3.12E-03	0.47
**RA**	rv0757	5	77.7	4.87E-03	0.72
**RA**	LPSSRH02C11	3	NA	7.88E-03	0.36
**EL**	LM15	NA	NA	2.18E-03	0.32
**EL**	LPSSRH01H06	4	43	2.26E-03	0.44
**EL**	rye012	4	53	2.83E-03	0.12
**EL**	LpHCA17C6	5	NA	6.84E-03	0.74

NA, not known

## Discussion

Plant heat tolerance is a complex trait that could be attributed by many phenotypic and physiological factors, including those parameters examined in this study, such as Fv/Fm, Pn, Chl content, WUE, and RWC, and root activity [9, 10, 22–25]. PCA is one effective strategy to integrate many factors reflecting facets of a complex trait and evaluate contribution of each factor to the trait, which has been successfully adopted in stress tolerance evaluation in soybean (*Glycine max*. L.) and switchgrass (*Panicum virgatum* L.) [26–28]. In this study, we applied the same strategy to integrate multiple parameters to rank heat tolerance among 98 accessions of perennial ryegrass. Furthermore, through Pearson correlation analysis, we found that EL, Chl content, and RWC were closely linked to overall heat tolerance ranking based on PCA. Unlike EL and RWC, Chl content could be quickly quantified using a chlorophyll meter. Therefore, Chl content alone or combined with EL could be used as the most convenient selection criterion for a large population of perennial ryegrass germplasm for elite heat tolerance.

Among heat tolerance-related morpho- and physiological traits, loss of chlorophyll is one hallmark of heat stress damages in cool-season grass species. Our previous studies found that leaf senescence induced by heat stress was mainly due to heat accelerated Chl catabolism rather than attenuated Chl biosynthesis in one cool-season grass, creeping bentgrass (*Agrostis stolonifera*) and reducing Chl catabolic rate by suppressing a Chl catabolic gene (*PPH*) in perennial ryegrass (*Lolium perenne* L.) delayed heat-induced leaf senescence [25, 29, 30]. Selecting for stay-green traits by controlling chlorophyll loss or leaf senescence is of great significance for improving cool-season grass species. Based on the finding that Chl content was associated with ryegrass heat tolerance, we checked the expression levels of *CCG*s in five most heat-tolerance and five heat-sensitive accessions. And indeed expression levels of several *CCG*s were strongly correlated to the corresponding heat tolerance ranking of each ryegrass accession. Thus, we considered that Chl content and Chl catabolic genes were reliable physiological trait and marker genes for evaluation of heat tolerance in perennial ryegrass accessions.

One recent report by Shin et al. (2020) showed that the natural variation within the promoter region of a Chl catabolic gene, *OsSGR*, was a predominant genetic factor for delayed leaf senescence in rice, and introgression of the promoter region of *OsSGR* from *Japonica*-type rice to *Indica*-type rice lead to delayed leaf senescence rate and up to 12.7% increased grain yield in the *Indica*-type rice [31]. The study by Shen et al. (2020) showed the potential of making use of the natural variation of CCGs [31]. Findings in this study showed that there are rich natural variations of these CCGs in terms of their responses to heat tolerance, and CCGs could also be used as potential target genes and/or markers for genetic improvement of ryegrass through gene editing or maker-assisted breeding.

Barre et al. (2017) classified 213 perennial ryegrass accessions into three clusters according to their vegetative and reproductive investment traits (e.g. leaf growth parameters, tillering parameters, heading data and reproductive investment) [32]. In this study, the 98 accessions of perennial ryegrass were classified into three clusters according to clustering analysis using 66 pairs of SSR markers that have been used to identify alleles contributing to plant tolerance to salt, drought, submergence, and winter stress, as well as spring re-growth [16, 18, 19]. Physiological traits positively correlated to heat tolerance, including Chl content, WUE, and RA had significantly greater levels in accessions in cluster C than those in cluster A and B based on SSR marker classification, suggesting that accessions in cluster C were more heat tolerant than those in cluster A and B, and variations in heat tolerance were related to their genetic structure in the population of perennial ryegrass examined in this study. It is also interesting to note that there is little evidence that these genetic clusters were related to their geographic location. Such a result could be interpreted by human activities’ disturbance in the dispersion and distribution of perennial ryegrass.

SSR markers linked with important agronomic traits, e.g. crown rust resistance [15], submergence [18], and heading date [33] in perennial ryegrass have been identified. However, no genic SSR markers that are associated with heat tolerance in perennial ryegrass have been reported so far. In the present study, twenty-nine SSRs linked to heat tolerance-related traits were detected in perennial ryegrass. Two markers LPSSRH01A07 and LPSSRH02C11, both located on chromosome 4, which were reported to be associated with relative growth rate under submergence stress [18], were correlated with WUE and RA in this study, suggesting a QTL in chromosome 4 could also affect heat tolerance. It is interesting to find that three markers M4213, B3B7, and PR37, located on chromosome 1 with 6 cM apart, were linked to WUE. In addition, Lp165, DLF008, and B3C10 located on chromosome 7 with 12 cM apart, were associated with Fv/Fm. Thus, it is most likely that a QTL for WUE and Fv/Fm located at chromosome 1 and 7, respectively. The results indicate that at least three candidate QTLs located in these chromosome positions play an important role in heat tolerance of perennial ryegrass.

## Conclusion

In summary, heat tolerance varied widely among 98 accessions of perennial ryegrass examined in this study. The most and least heat-tolerant accessions were identified, and accessions in cluster C were relatively more heat tolerant than those in cluster A and B. EL, Chl content and CCGs were reliable physiological traits and marker genes for heat stress tolerance assessment in perennial ryegrass. Furthermore, SSR markers associated with Chl content, Fv/Fm, Pn, WUE, EL, and RA were also identified. The result highlighted the importance of cell membrane stability and Chl catabolism in heat tolerance of cool-season grasses. Such knowledge is of significance for heat-tolerance breeding of perennial ryegrass and for further studies on heat tolerance mechanisms in perennial ryegrass as well as in other cool-season grass species.

## Methods

### Plant materials and growth conditions

A collection of 98 perennial ryegrass accessions was obtained from United States Department of Agriculture’s National Plant Germplasm System (USDA-GRIN), including 37 wild, 33 cultivated, and 28 with uncertain pedigree accessions (Table 6). A single seed of each germplasm was sown in plastic pots (13 cm diameter and 13 cm height) filled with fritted clay and maintained in a greenhouse at Nanjing Agricultural University, Jiang Su, China. Each accession was propagated using tillers to generate stock plants. In this experiment, tillers from stock plants of each accession were transplanted into eight pots (10 tillers in each pot) and maintained in a growth chamber controlled at 25/20 °C (day/night temperature), 70% relative humidity, a photoperiod of 16 h, and photosynthetic active radiation of 750 μmol m^− 2^ s^− 1^ for 60 d. Plants were maintained at a height of 12 cm by weekly mowing and fertilized weekly with half-strength Hoagland’s nutrient solution [34].

**Table 6:** Accession number (PI No.), origin, status, and genetic clusters of 98 perennial ryegrass accessions

Accession No.	Origin	Status^a^	Cluster	Accession No.	Origin	Status^a^	Cluster
**287,855**	Spain	U	A	284,823	Australia	U	C
**231,606**	Portugal	U	A	231,604	Portugal	U	C
**610,950**	Morocco	W	A	516,605	Yugoslavia	CD	C
**220,178**	Afghanistan	W	A	371,952	Bulgaria	U	C
**265,340**	Portugal	U	A	440,474	Former USSR	W	C
**319,018**	Spain	U	A	265,351	Chile	U	C
**410,155**	South Africa	W	A	231,576	Algeria	U	C
**577,251**	Morocco	W	A	418,726	France	W	C
**231,566**	Libya	U	A	403,868	Canada	C	C
**287,849**	Spain	U	A	231,580	Algeria	U	C
**239,730**	Egypt	U	A	634,205	USA	C	C
**206,376**	Cyprus	U	A	198,070	Sweden	CD	C
**268,333**	Former USSR	W	A	321,681	France	C	C
**423,136**	Spain	W	A	266,293	Netherlands	CD	C
**182,857**	Czech Republic	U	B	578,763	USA	C	C
**538,976**	Russia	C	B	321,397	Czech Republic	U	C
**197,270**	Finland	CD	B	303,020	Sweden	C	C
**547,390**	Iran	W	B	231,595	Morocco	U	C
**204,085**	Cyprus	U	B	418,721	Belgium	W	C
**619,474**	Romania	CD	B	418,722	Luxembourg	W	C
**306,292**	Bolivia	U	B	418,741	France	W	C
**277,848**	Cyprus	U	B	619,003	Norway	W	C
**231,567**	Libya	U	B	251,224	Yugoslavia	W	C
**598,911**	Tunisia	W	B	628,717	Bulgaria	W	C
**598,839**	Morocco	W	C	290,368	Hungary	U	C
**418,712**	Yugoslavia	U	C	376,878	New Zealand	C	C
**277,846**	Yugoslavia	U	C	303,037	Sweden	C	C
**610,802**	Norway	W	C	420,126	Japan	U	C
**231,565**	Libya	U	C	204,880	Turkey	W	C
**225,825**	Denmark	U	C	265,344	Ireland	C	C
**204,879**	Turkey	W	C	237,187	Netherlands	CD	C
**265,342**	Ireland	C	C	577,254	Luxembourg	W	C
**303,026**	France	C	C	274,637	Poland	U	C
**275,660**	Australia	CD	C	298,091	Hungary	W	C
**418,723**	Luxembourg	W	C	285,101	Australia	C	C
**577,273**	Turkey	W	C	505,842	Former USSR	CD	C
**577,269**	Norway	W	C	632,542	Hungary	C	C
**238,938**	New Zealand	U	C	610,965	Italy	W	C
**267,059**	Poland	U	C	202,451	Argentina	W	C
**303,027**	Denmark	C	C	231,605	France	C	C
**317,452**	Afghanistan	W	C	284,826	Australia	U	C
**403,907**	Canada	C	C	502,412	Russia	W	C
**278,773**	Canada	C	C	418,714	Italy	W	C
**254,898**	Iraq	W	C	598,434	Italy	W	C
**267,058**	Poland	U	C	610,926	Tunisia	W	C
**418,736**	Switzerland	W	C	420,128	Japan	C	C
**598,443**	Switzerland	W	C	418,707	Romania	W	C
**600,878**	USA	C	C	598,892	Tunisia	W	C
**303,028**	Denmark	C	C	311,075	Romania	U	C

Status^a^: Improvement status obtained from USDA germplasm bank

*U* uncertain, *W* wild, *C* cultivar, *CD* cultivated

### Temperature treatments

Plants in four pots (four replicates) for each accession were exposed to normal growth temperature of 25/20 °C (day/night) or heat stress at 35/30 °C (day/night) in growth chambers. Each temperature treatment was repeated in two growth chambers. Plants were subjected to those temperature conditions for 24 d. The plants received regular water and fertilization during the treatment period.

### Phenotypic and physiological measurements

For leaf width (LW) analysis, 10 mature leaves of each accession were measured using a digital caliper. Maximum plant height (PH) was measured manually as the length from the base to the top of each plant. TQ was visually rated using a 1–9 scale based on plant color, shoot density and uniformity to assess the overall plant health and vigor (1 represents brown and dead plant, and 9 represents the best plant in all these quality components) [35]. Fv/Fm was determined using a fluorescence meter (Dynamax, Houston, TX, USA), described previously by Oxborough and Baker (1997) [36]. Chl content was measured according to the method by Arnon (1949) [37]. For RWC quantification, about 0.1 g fresh leaves were detached and immediately weighed as the fresh weight (FW), then soaked in distilled water and maintained at 4 °C in the dark for 24 h and weighted as turgid weight (TW). Leaf samples were then placed in an oven at 80 °C for 72 h prior to being weighted for dry weight (DW). The RWC was calculated as formula: (FW-DW)/(TW-DW) × 100% [38]. Pn and WUE were measured according to the method described by Burgess and Huang (2014) using a portable photosynthesis system (Li-COR6400, LI-COR Inc., Lincoln, NE, USA) [39]. For quantification of EL, approximately 0.2 g of leaves were collected, rinsed three times with distilled water, and then placed into a 50 ml tubes containing 30 ml distilled water. Initial conductivity (Ci) was quantified using a conductivity meter (YSI Model 32, Yellow Spring, OH) after shaking for 24 h. The samples were then autoclaved for 30 min. The maximum conductivity (Cmax) was measured after the tubes were cooled to room temperature. The relative EL was calculated as (Ci/Cmax) × 100.

RA was measured using 2,3,5-triphenyl tetrazolium chloride (TTC) reduction method [40]. The TTC reduction assay was performed following the method of Steponkus and Lanphear (1967) with minor modifications [41]. In brief, approximately 0.5 g root tips (0–50 mm) were excised and washed three times with distilled water and transferred to 10 ml TTC solution (0.4% TTC in 0.1 M sodium phosphate buffer, pH 7.0). After 3 h incubation at 37 °C, TTC solution was removed and root segments were washed with distilled water. The roots were cut into 1 cm segments and incubated overnight at room temperature in 10 ml of 95% ethanol. The reduction of TTC was expressed as the absorbance of the extraction solution at 485 nm.

### Ranking of overall heat tolerance

Principal component analysis (PCA) was used to rank heat tolerance of 98 accession, following the method used in ranking of drought and salinity tolerance for different grass genotypes as described in Liu et al. (2015) [27] and Tang et al. (2013) [19]. PCA ranking value for each accession was calculated using the formula: PCA rank value = ${\sum}_{j=1}^n\left[ PCj\times contribution\ of\  PCj\left(\%\right)\right]\ j=1,2,3,\dots, n$ [42]. In this formula, ‘PCj’ represents the value of principal component *j* and ‘contribution of PCj (%)’ represents the variance in response to stress treatment that principal component *j* could explain. When the total contribution of first several PCs was higher than 85%, these PCs were selected for PCA rank calculation [43]. Relative heat tolerance of 98 perennial ryegrass accessions was subsequently ranked according to their PCA ranking values.

The absolute value of each physiological value across different accessions cannot faithfully represent the degree of their heat tolerance. For example, an accession with low Chl content after heat stress cannot tell whether this low content was due to the stress or due to other factors (e.g. it might be low even without heat stress). Therefore, we used a relative value, Heat stress index (HSI), for the stress-tolerance evaluation [26]: HSI = (value of parameter under heat stress condition) / (value of parameter under control condition) × 100.

### Genetic diversity and association analysis

A total of 66 pairs of publicly available SSR primers of nuclear DNA distributed on seven chromosomes (Supplementary materials 3) were used to genotype the 98 perennial ryegrass accessions [19]. PCR reaction was performed in a 10 μl reaction volume with 60 ng DNA, 1.0 unit of *Taq* DNA Polymerase, 1 × PCR buffer, 0.2 mM dNTP mix, 2.5 mM MgCl_2_, 0.05 μM forward tailed primer, 0.05 μM fluorescent labeled M13 primer, and 0.1 μM reverse primer. All PCR reactions were carried out in a Bio-Rad thermocycler (Bio-Rad Inc., Hercules, CA, USA) using a touch-down program described by Yu et al. (2013) [16]. PCR products were separated in an ABI 3730 DNA Sequencer (Applied Biosystem, Inc., Foster City, CA, USA). Alleles were identified by GeneMarker 1.6 software (SoftGenetics, LLC, State College, PA, USA). The allelic bands with at least 2 bp differences were considered as polymorphic among accessions. All confirmed polymorphic alleles were used for cluster analysis.

Nei’s genetic distance among accessions was estimated by the “Phylogeny” function of PowerMarker software version 3.25 based on the SSR marker data [44]. The Nei’s genetic distance was then used for cluster analysis and generation of Neighbour Joining (N-J) dendrogram by FigTree version 1.4.2 (http://tree.bio.ed.ac.uk/software/figtree/) [45]. Association analysis of SSR markers with heat tolerance traits was conducted using TASSEL 5.0 software along with the general liner model (GLM) procedure [46]. *P* ≤ 0.01 was used to identify significant associations.

### Gene expression analysis

The Qiagen RNA Extraction Kit (Qiagen, Valencia, CA, USA) was used to extract total RNA form perennial ryegrass leaves following the manufacturer’s instructions. The first-strand cDNA was synthesized using the High Capacity cDNA Reverse Transcription Kit (Applied Biosystems, Grand Island, NY, USA). The qRT-PCR reaction was performed using the Roche LightCycler® 480 II Real-Time PCR System (Roche Molecular Systems, Inc., Branchburg, NJ, USA). All PCR reactions were performed in a 20 μl reaction volume with four biological replicates. Primers used for qRT-PCR analysis were listed in supplementary Table 3 and *LpeIHF4A* was used as a reference gene [47].

### Data analysis

Data from all samples were subjected to analysis of variance (ANOVA) using SAS 9.3 software (SAS Institute Inc., Cary, NC, USA) as shown in Table 1. Treatment means were separated using Fisher’s protected LSD test at a 0.05 probability level as shown in Table 4 and Fig. 4. Pearson correlation analysis between physiological parameters was performed using the Bivariate Correlations program in software SPSS for Windows (Version 12, SPSS Inc., Chicago, IL) as shown in Table 3. Heatmap (Fig. 1) and principal component analysis (PCA) (Fig. 2) of the morphological and physiological parameters were performed using R statistical software (R3.5.2 by R Development Core Team).

## Supplementary information

**Additional file 1: Table S1.** Eigenvectors and percentage of accumulated contribution ratios of each principal component. **Table S2.** Principle component analysis of eight physiological traits across 98 perennial ryegrass accessions using heat stress index (HSI) after 24 days of treatment. **Table S3.** Primers used for gene expression analysis.

**Additional file 2: Supplementary materials 1.** Values of all physiological traits before and after heat tolerance in 98 ryegrass accessions.

**Additional file 3: Supplementary materials 2.** Major allele frequency, polymorphic bands, polymorphism information content, and gene diversity index of 66 pairs of SSR used for genetic diversity analysis in 98 ryegrass accessions.

**Additional file 4: Supplementary materials 3.** Linkage group, physical distance, and primer sequences of 66 SSRs used in this study.

## Data Availability

The data sets and materials supporting the findings of this article are presented within the manuscript and its supplementary information files.

## Abbreviations

Chl: Chlorophyll
CCGs: Chl catabolic genes
TQ: Turf quality
Fv/Fm: Photochemical efficiency
RWC: Leaf relative water content
EL: Electrolyte leakage
Pn: Photosynthetic rate
WUE: Water use efficiency
RA: Root activity
LW: Leaf width
PH: Maximum plant height
PCA: Principal component analysis
HIS: Heat stress index
SSR: Simple sequence repeat
