# Supplementary material for: Natural variation of physiological traits, molecular markers, and chlorophyll catabolic genes associated with heat tolerance in perennial ryegrass accessions
Source: BMC Plant Biol. 2020 Nov 16;20:520. doi: 10.1186/s12870-020-02695-8 (PMC7667755; doi:10.1186/s12870-020-02695-8)
Supplement: Supplementary file 1 [file 12870_2020_2695_MOESM1_ESM.docx]

Supplementary table 1. Eigenvectors and percentage of accumulated contribution ratios of each principal component. 

| Primers | Target genes’ Accession no. | Sequence (5'-3') |
| --- | --- | --- |
| LpNYC1-QRTF | KX686491 | GATCGTCTCCCAGAAGTGCT |
| LpNYC1-QRTR | KX686491 | GCCAGTCTCCTGCTTGAAC |
| LpNOL-QRTF | KX686493 | GCTGGCAAAGAAGTTTCTCA |
| LpNOL-QRTR | KX686493 | ATGCTGCTCTCCAAATTCCT |
| LpPPH-QRTF | KT345726 | ACCCAGGTGATTCAGGAAAG |
| LpPPH-QRTR | KT345726 | CCTGACCTCACCAACCTTCT |
| LpSGR-QRTF | KX791203 | GAGGAGGCGAACTCGAAG |
| LpSGR-QRTR | KX791203 | GGTTGTACCACCCTTGCAG |
| LpelF4A-QRTF | GO924770 | AACTCAACTTGAAGTGTTGGAGTG |
| LpelF4A-QRTR | GO924770 | AGATCTGGTCCTGGAAAGAATATG |

Supplementary table 2. Principle component analysis of eight physiological traits across 98 perennial ryegrass accessions using heat stress index (HSI) after 24 days of treatment.

| Principal component | Initial Eigenvalues |
| --- | --- |
| Eigenvalue | Contribution ratio (%) | Cumulative contribution ratio (%) |
| PC1 | 3.80 | 54.34 | 54.34 |
| PC2 | 1.63 | 23.25 | 77.59 |
| PC3 | 0.61 | 8.69 | 86.28 |
| PC4 | 0.30 | 4.25 | 90.53 |
| PC5 | 0.27 | 3.84 | 94.36 |
| PC6 | 0.26 | 3.76 | 98.12 |
| PC7 | 0.13 | 1.88 | 100.00 |

Supplementary table 3. Primers used for gene expression analysis.

| Traits | PC1 | PC2 | PC3 | PC4 |
| --- | --- | --- | --- | --- |
| TQ | 0.897 | -0.199 | 0.196 | 0.031 |
| Fv/Fm | 0.766 | -0.311 | 0.413 | -0.125 |
| Chl | 0.858 | -0.055 | -0.119 | 0.219 |
| Pn | 0.373 | 0.809 | 0.233 | -0.220 |
| WUE | 0.244 | 0.896 | 0.033 | 0.130 |
| EL | -0.691 | -0.043 | 0.566 | 0.431 |
| RWC | 0.891 | -0.180 | 0.095 | -0.166 |
| RA | 0.785 | 0.040 | -0.227 | 0.480 |
| CR | 54.34 | 23.25 | 8.69 | 4.25 |

PC1, the first principle component; PC2, the second principle component; PC3, the third principle component; PC4, the fourth principle component; CR, Contribution ratio.
